# Shells as ‘extended architecture’: to escape isolation, social hermit crabs choose shells with the right external architecture

**DOI:** 10.1007/s10071-020-01419-7

**Published:** 2020-08-08

**Authors:** Jakob Krieger, Marie K. Hörnig, Mark E. Laidre

**Affiliations:** 1 Department of Cytology and Evolutionary Biology, Zoological Institute and Museum, University of Greifswald, Soldmannstraße 23, 17489 Greifswald, Germany; 2 Department of Biological Sciences, Dartmouth College, Hanover, NH 03755 USA

**Keywords:** Architecture, Arthropods, Extended architecture, Extended phenotypes, Invertebrates, Physical cognition, Problem solving, Shells, Social cognition, Spatial cognition

## Abstract

Animals’ cognitive abilities can be tested by allowing them to choose between alternatives, with only one alternative offering the correct solution to a novel problem. Hermit crabs are evolutionarily specialized to navigate while carrying a shell, with alternative shells representing different forms of ‘extended architecture’, which effectively change the extent of physical space an individual occupies in the world. It is unknown whether individuals can choose such architecture to solve novel navigational problems. Here, we designed an experiment in which social hermit crabs (*Coenobita compressus*) had to choose between two alternative shells to solve a novel problem: escaping solitary confinement. Using X-ray microtomography and 3D-printing, we copied preferred shell types and then made artificial alterations to their inner or outer shell architecture, designing only some shells to have the correct architectural fit for escaping the opening of an isolated crab’s enclosure. In our ‘escape artist’ experimental design, crabs had to choose an otherwise less preferred shell, since only this shell had the right external architecture to allow the crab to free itself from isolation. Across multiple experiments, crabs were willing to forgo preferred shells and choose less preferred shells that enabled them to escape, suggesting these animals can solve novel navigational problems with extended architecture. Yet, it remains unclear if individuals solved this problem through trial-and-error or were aware of the deeper connection between escape and exterior shell architecture. Our experiments offer a foundation for further explorations of physical, social, and spatial cognition within the context of extended architecture.

**Electronic supplementary material:**

The online version of this article (10.1007/s10071-020-01419-7) contains supplementary material, which is available to authorized users.

## Introduction

Animal minds have been shaped by natural selection to solve re-occurring social and ecological challenges faced in the wild over the course of their species’ evolutionary history (Bueno-Guerra and Amici 2018). Such challenges in nature ultimately favored cognition (Bayne et al. 2019), which allows individuals to acquire, process, store, and act on information (Shettleworth 2010), thereby navigating their environment and making adaptive decisions that enhance their Darwinian fitness. Specialized forms of cognition allow animals to solve recurrent problems, which were faced again and again over their species’ evolutionary history (Shettleworth 2010). However, a hallmark feature of higher-order cognition is the ability to solve novel problems (Bayne et al. 2019), which extend beyond what the species experienced in the past (over its evolutionary history) and which also extend beyond what any individual might previously have encountered (over its lifetime). Problems like this are effectively ‘doubly novel’ (Gould and Gould 1994; Heinrich 1995; Griffin 2001), since neither the individual (over its ontogeny) nor the species (over its phylogeny) have confronted them before. Ultimately, if scientists are to fully map the extent of cognitive skills across the animal kingdom, it is essential we devise experiments that (1) are grounded in the fundamental ecological and evolutionary ingredients of a species’ natural history (Bueno-Guerra and Amici 2018) and (2) simultaneously challenge the upper limits of cognitive abilities using novel experimental designs (de Waal 2017).

Experiments on navigation have provided a powerful means of testing animal cognition (e.g., Vannini and Cannicci 1995; Zeil and Hemmi 2006; Wehner 2020). For example, studies spanning both invertebrates and vertebrates have tested individuals’ ability to navigate from novel release points and choose between alternative routes home. Many of these studies have revealed that individuals can choose the shortest and most effective routes back home, both over short and long distances (reviewed in Gould and Gould 2012). However, while most animals must navigate solely with consideration of their own body passing through space (e.g., Lenkei et al. 2020), some animals have an even greater navigational challenge: moving with a physical extension of their body, such as an architectural object they temporarily carry, which reaches beyond their own body and effectively alters the extent of physical space that the individual occupies in the world. Here, we term such architectural extensions of an organism’s body ‘extended architecture’, in deference to Dawkins’ (1982) concept of ‘extended phenotypes’. Extended architecture presents a unique opportunity for testing cognitive abilities (cf. Japyassú and Laland 2017), especially on novel problems: given variation in the physical form of extended architecture, individuals may need to substantially alter their strategies for escaping, particularly once they are faced with novel barriers to their movement and a choice between different forms of extended architecture.

Among nonhuman animals, hermit crabs (Decapoda, Anomura) represent one of the few that roam with extended architecture, as represented by their shell (Vermeij 1993). The shell is not part of the crab’s own body, but the crab’s choice of which shell to occupy is influenced by its genes and thus parallels the concept of an ‘extended phenotype’ (sensu Dawkins 1982). Aside from one species of hermit crab (adults of *Birgus latro*; Drew et al. 2010; Krieger et al. 2012a; Laidre 2018a), every one of the approximately 1000 species of hermit crab worldwide carries a shell as it navigates (Hazlett 1981; Laidre 2011). Some hermit crab species even use coconuts as shells (Laidre 2019a; cf. Finn et al. 2009 in octopus), rather than the more typical seashells that are left over from dead or predated snails (Valdes and Laidre 2018). Irrespective of its origin, the shells used by hermit crabs are all external objects and thus distinct from the crab’s own body. And while a crab may switch shells, the crab always carries its current shell with it as it navigates the surrounding environment, since doing so ultimately serves an adaptive function of providing an externally derived form of cover and a portable home, thereby increasing survival and reproductive success. Shell use is almost certainly ‘hard wired’ in hermit crabs (Hazlett 1981), but intriguing evidence suggests that cognition underpins how crabs navigate with this extended architecture. For example, when hermit crabs switch shells, they exhibit an awareness of the resulting change in their ‘virtual body’ (Sonoda et al. 2012), updating on how they move around impediments based on the variable external architecture (e.g., spines and other extensions) of their changed shell. Building on such a cognitive foundation (Elwood and Neil 1992), future experiments could generate important insights into cognitive abilities while navigating with extended architecture by testing hermit crabs on novel problems—ones which challenge these animals to choose shells that solve novel escape dilemmas they have never previously encountered.

Here, we conducted a series of experiments that posed a novel problem for these invertebrates: choose the right shell to escape solitary confinement. Our experiments focused on a highly social terrestrial hermit crab species (*Coenobita compressus*). These ‘social hermits’ (Laidre 2014) not only carry shells as they navigate, but also modify shells by architecturally remodeling their interior (Laidre 2012a), which makes the shells highly valuable (Laidre 2019b) and better suit them to a terrestrial lifestyle (Laidre et al. 2012). Architecturally remodeled shells thus qualify as ‘extended phenotypes’ (sensu Dawkins 1982), and the remodeling process itself has had major evolutionary consequences for this species, making it highly social (Laidre 2012b), due to a dependency among conspecifics for remodeled shells (reviewed in Laidre 2018b).We built on this ecological and evolutionary foundation of sociality and sophisticated agility with extended architecture to provide these animals with a novel escape problem, which they had never encountered before: we asked them to choose between two shells, only one of which would allow them to escape solitary confinement, because only that shell’s external architecture matched the escape opening. In our ‘escape artist’ experimental design, we tested individuals by confining them alone in an enclosure in the wild, where they would have a strong motivation to get out—for only by escaping social isolation could they subsequently be around conspecifics and thereby access critical resources, including better shells (Laidre 2010). Furthermore, we made this problem extra challenging by having the ‘correct’ shell (which was needed to escape confinement) also be the shell that was less preferred in baseline control conditions. Hence, to solve this novel problem, individuals had to alter their preferred shell choice.

## Material and methods

### Study site and species

**Fig. 1:**
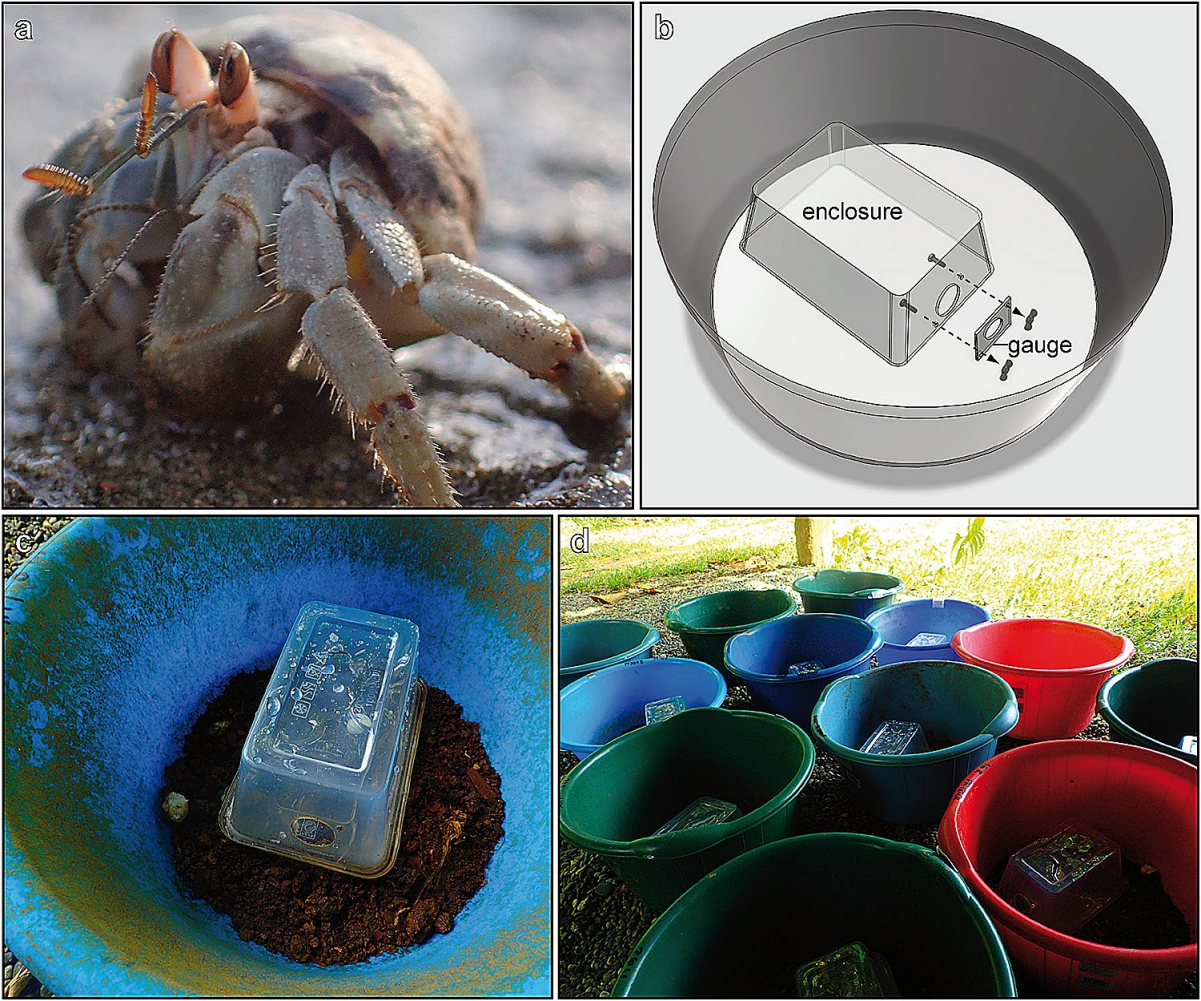
‘Escape artist’ experimental design. Each individual was isolated within an enclosure, and only by choosing the correct shell could it escape solitary confinement and rejoin conspecifics in the wild. **a** Photo of a social hermit crab (*Coenobita compressus*), the study species. **b** Schematic of the isolated enclosure setup, including the mountable gauge, which only allowed specific shells to fit through the escape opening during the experimental condition. Photos of **c** a single enclosure and **d** many enclosures, each in separate buckets, arrayed in the field

Experiments were conducted during February and March 2018 in Osa Peninsula, Costa Rica, at a long-term field site (Osa Conservation’s Piro Biological Station), where the *C. compressus* population has been under study in the wild since 2008 (Laidre 2010). Individuals of this species (Fig. 1a) roam on land across open sandy beaches and within forests (Laidre 2013a), using a wide variety of shell species (Laidre and Vermeij 2012), each with variable exteriors, shapes, and sizes, thus representing different extended architecture. Individuals frequently navigate around various natural impediments, like trees and rocks. Sometimes, individuals can also be caught and die within hazardous plastic debris, which has washed up on the beach and is impossible to escape (Lavers et al. 2020). But never in over a decade of study (Laidre, personal observation) and never, to our knowledge, over this species’ evolutionary history (Laidre 2018b) have individuals experienced the type of navigational challenge we posed to them in our experiments. Our experiments thus represented a novel problem.

### Novel navigational problem: choosing the correct shell to escape

In our ‘escape artist’ experimental design, we tested if individuals could solve a novel problem: escaping solitary confinement by choosing the correct shell of two alternatives. Crabs were collected directly from the wild and placed individually into an enclosure (Fig. 1b), which had only a single opening through which the individual could escape. As extended architecture, we provided artificial 3D printed shells (see below), which the crabs readily moved into and utilized. 3D printing allowed us to design shells such that only one was the right match (in terms of external architecture) to fit through the escape opening of an isolated individual’s enclosure. Across all experiments, the correct shell needed to escape was the one that was otherwise less preferred by crabs in baseline control conditions. Thus, if an individual was to successfully navigate out of confinement, freeing itself from isolation to rejoin conspecifics in the wild, it had to forgo the preferred shell and choose the less preferred shell.

### Enclosures

Enclosures (*N* = 40) were rectangular plastic containers (155 × 102 × 74 mm), fully sealed with a lid. On one side of each enclosure, we drilled a hole (28 mm diameter) as an escape opening (Fig. 1b, c). On top of this escape opening, we attached a custom-made PVC-plate gauge (51 × 37 × 2 mm), using two metric machine screws (ISO size M4 × 10 mm) with wingnuts. Cut into the gauge was an ellipsoid shape (1.6 mm in height and 1.9 mm in width), which acted as a filter for what shells could pass through the opening. In experimental conditions, we attached the gauge, which allowed only specific shells (see below) to transit through the escape opening. In contrast, in control conditions, we removed the gauge, which allowed any and all shells to transit through the escape opening. Each enclosure was also placed within a large plastic bucket (25 cm base diameter) filled with a layer of humid soil (Fig. 1c), allowing us to monitor many individuals in the wild simultaneously (Fig. 1d) and determine whether they were able to escape their enclosure.

### Extended architecture: 3D printed shells

**Fig. 2:**
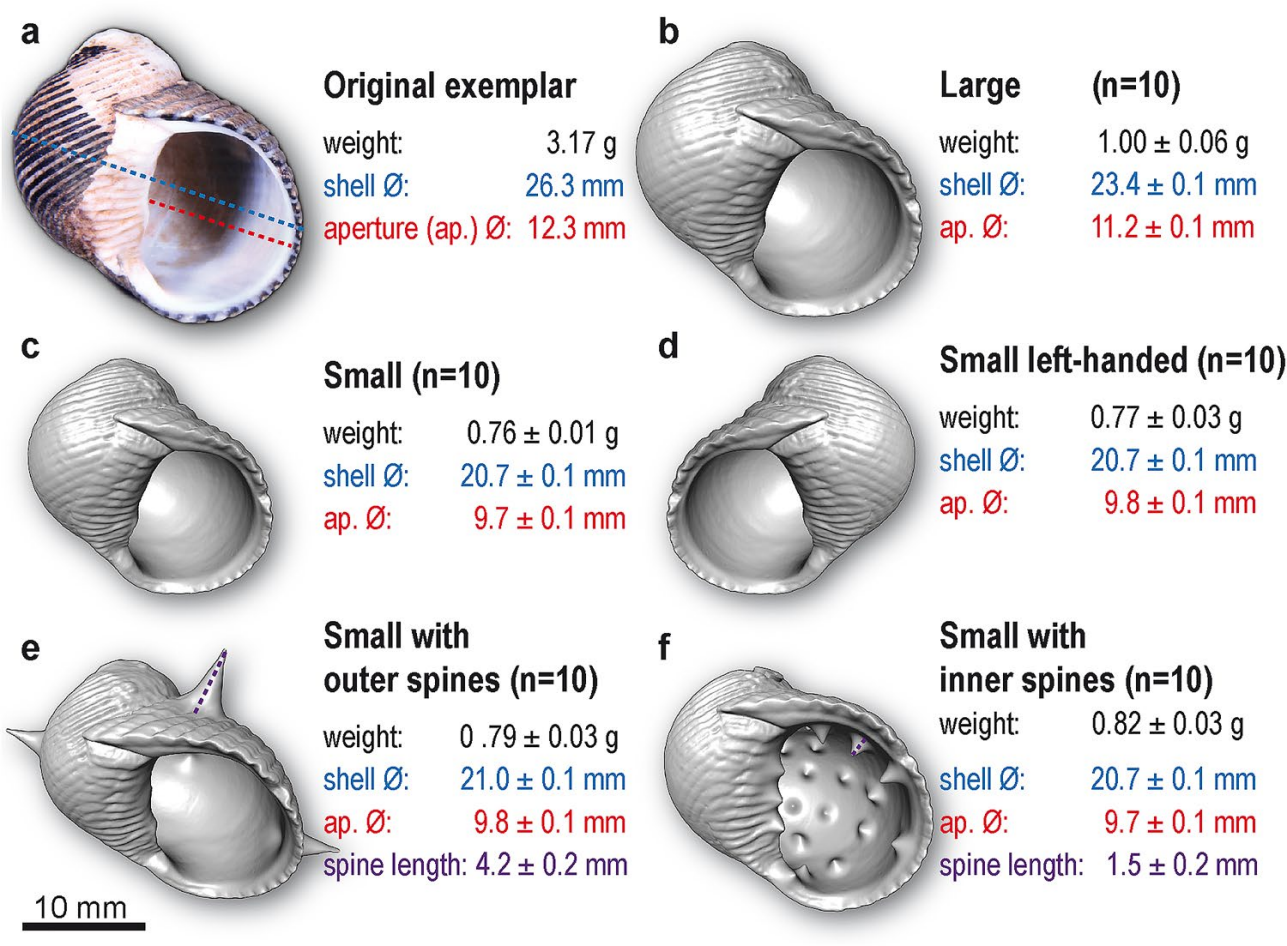
Shells as ‘extended architecture’. **a** The original exemplar (an architecturally remodeled *Nerita scabricosta* shell), which is the shell most preferred by social hermit crabs (*Coenobita compressus*). This natural shell (composed of calcium carbonate) was scanned (µCT) and then served as the basis for the following five artificial shell types, which were 3D printed (in plastic) and used as ‘extended architecture’ in the escape experiments. **b** The ‘large’ type was 90% of the size of the original exemplar. **c** The ‘small’ type was 80% of the size of the original exemplar. Three other shell types were identical in size to the ‘small’ type, except with additional alterations to their architecture. **d** The ‘left-handed’ type was sinistral (i.e., left-handed), instead of the near-universal dextral (i.e., right-handed) shape. **e** The ‘outer spines’ type had spines on its exterior surface. **f** The ‘inner spines’ type had spines on its interior surface. Standardized architectural measures (mean ± SE) are shown for *N* = 10 specimens of each type

All 3D printed shells were made based on an original exemplar (Fig. 2a), a natural shell, which was one of the architecturally remodeled *Nerita scabricosta* shells known to be highly preferred by the crabs (Laidre 2012a). This exemplar was imaged at the University of Greifswald with a laboratory-based 3D X-ray microscope (µCT) XRadia MicroXCT-200 (Carl Zeiss Microscopy GmbH). It was scanned at 0.4 × optical magnification at a voltage of 90 kV and a power of 88 µA using an X-ray exposure of 1 s per projection, resulting in 1600 single projections covering a full rotation of the specimen. Reconstruction of the tomography was performed using XMReconstructor (Carl Zeiss Microscopy GmbH), generating scaled image stacks (DICOM format). The noise of the scan was reduced by summarizing four pixels into one (“binning 2”), while the subsequent reconstruction was performed at full resolution (“binning 1”) to avoid information loss, yielding a voxel size of 31 µm. The resulting image stacks were then imported into the 3D software Amira 5.4.1 (FEI), converted into a 3D-mesh file (STL-format) using the modules “Isosurface” and “Extract Surface”, smoothed using the module “Smooth surface”. By using the modules “Label field” and “SurfaceGen”, the internal volume as well as the area of aperture was manually labeled and measured using the module “Surface Area”. The resulting mesh was finally reduced to a total of 600,000 triangles to obtain a more manageable file size in advance of printing.

Five different ‘extended architecture’ shell types (Fig. 2b–f) were produced. The ‘large’ type (Fig. 2b) was 90% of the size of the original exemplar. The ‘small’ type (Fig. 2c) was 80% of the size of the original exemplar. The three other shell types were identical in size to the ‘small’ type, except with additional alterations to their architecture, which we made using the open source software Meshmixer 3.4.35 (Autodesk, Inc. 2017). Specifically, the ‘left-handed’ type (Fig. 2d) was sinistral (i.e., left-handed), instead of the near-universal dextral (i.e., right-handed) shape. The ‘outer spines’ type (Fig. 2e) had spines on its exterior surface. And the ‘inner spines’ type (Fig. 2f) had spines on its interior surface.

Of these five shell types, two (‘large’ and ‘outer spines’) were deemed incorrect shells, since they did not fit through the escape opening in the experimental conditions and thus could not be used to escape. Only the other three shell types (‘small’, ‘left-handed’, and ‘inner spines’) could be used to escape in the experimental conditions, so only these shell types were deemed correct shells. Critically, each of the three correct shells, by design, had elements that would make them less desirable to *C. compressus* individuals during baseline control conditions. In particular, individuals (of an appropriate reference body size) typically prefer large rather than small shells; individuals also prefer shells with the near universal right-handed curvature, not the unnatural and exceedingly rare left-handed curvature; and finally, individuals avoid shells with abrasive interior elements, like inner spines, which rub against crabs’ delicate hind body (pleon) (Laidre, personal observations). Thus, the correct shells, which were essential to escaping, were otherwise undesirable.

Models of each of the five shell types were imported into the open source 3D printing software CURA 3.0.3 and were printed using a white filament of polylactic acid 3 mm in diameter (NuNus PLA; KeyCoon GmbH and Kaisertech PLA, Berlin). Printing was performed using an Ultimaker 2 desktop-based 3D printer equipped with a 0.4 mm nozzle using the following parameters: layer height of 0.1 mm; wall thickness of 1 mm; infill density of 50%; printing speed of 60 mm per sec; and a travel speed of 100 mm per second (using retraction). The shells were printed using “zigzagging”-support structures (using a density of 15% and a line width of 2.67 mm) touching the build plate to provide a better adhesion during printing. After printing, we randomly selected *N* = 10 specimens of each of the five shell types and took standardized architectural measurements (see Laidre 2012a) of shell diameter, aperture (opening), weight, as well as spine length for those shells that had outer or inner spines. The final printed products across each different shell type represented quite distinct examples of extended architecture (Fig. 2b–f).

### Experiments 1–4

We conducted a total of four separate experiments, each experiment involving a different pair of shell types, which individuals had to choose between. In our experiments we only tested individuals whose original natural shells corresponded closely in size to the exemplar shell we had used to make the 3D printed shells. For each experiment, we first tested *N* = 40 individuals in baseline control conditions (in which the enclosure’s escape opening lacked a gauge, thus allowing individuals to readily move back-and-forth through this opening with either shell). We then tested a new set of *N* = 40 individuals in experimental conditions (in which the enclosure’s escape opening had a gauge, thus allowing escape only if individuals chose the correct shell). In each of the four experiments, only one of the two 3D printed shells represented the correct shell needed to escape during the experimental conditions:

- Experiment 1: ‘large’ vs. ‘small’ (‘small’ was correct).
- Experiment 2: ‘outer spines’ vs. ‘small’ (‘small’ was correct).
- Experiment 3: ‘outer spines’ vs. ‘left-handed’ (‘left-handed’ was correct).
- Experiment 4: ‘outer spines’ vs. ‘inner spines’ (‘inner spines’ was correct).

It is worth noting that *C. compressus* individuals can readily switch from one shell to another (Laidre 2019c), but that it is dangerous for individuals to remain naked (i.e., not occupying a shell) for more than a short period, since then they can desiccate and die (Laidre 2012b). Individuals in our experiments were therefore never observed trying to escape enclosures in a naked state. Furthermore, in our experiments, we ensured all individuals would remain safe, and not be at risk of desiccation, by doing the following: after gently removing individuals from their original natural shell,^1^ we did not place them into the enclosure naked, but instead immediately allocated them to one of the two 3D printed shells—specifically the incorrect one. Individuals thus began their trial in an incorrect shell, so they were safe from desiccation, but would not be able to escape their enclosure in that shell. To escape, they had to voluntarily switch shells and commit to choosing the correct shell, which we placed empty at the far corner of the enclosure, its aperture turned up.

For every trial across all experiments, we provided brand new 3D printed shells, which had never been used before. We also cleaned enclosures between trials, thus ensuring that every individual tested had to solve the problem independently (with no preexisting cues from conspecifics about the correct solution). Each individual (*N* = 320) was tested only once, in a single trial in which it was given up to 20 h. For the control condition, we recorded what shell each individual was occupying at the end of the 20 h, which established the crabs’ baseline ‘shell preference’(Elwood and Neil 1992) when there was no constraint on ‘escaping’ and when individuals could readily move back and forth through the enclosure opening using either shell. For the experimental condition, we recorded whether individuals were able to solve the problem of escaping by choosing the correct shell. And if they could not, then they were deemed unsuccessful. The individuals we tested were randomly collected from a population of hundreds of thousands of individuals (Laidre and Vermeij 2012). At the end of our experiments, we freed all tested individuals, first putting them back in their original natural shells and then releasing them unharmed into the wild.

### Prediction and statistical analyses

If individuals have the ability to solve this novel escape problem with extended architecture, then during the experimental conditions (relative to the baseline control conditions) they should be more likely to select the correct shell and thereby escape solitary confinement. To test this prediction, we conducted Chi-square tests for each of the four experiments, comparing the number of crabs that chose the correct rather than incorrect shell in experimental versus control conditions. In addition, we conducted two separate sets of Chi-square tests (see Table S1) that compared the number of crabs that chose the correct shell in the control condition as well as in the experimental conditions to an arbitrary threshold of 50%.

## Results

**Fig. 3:**
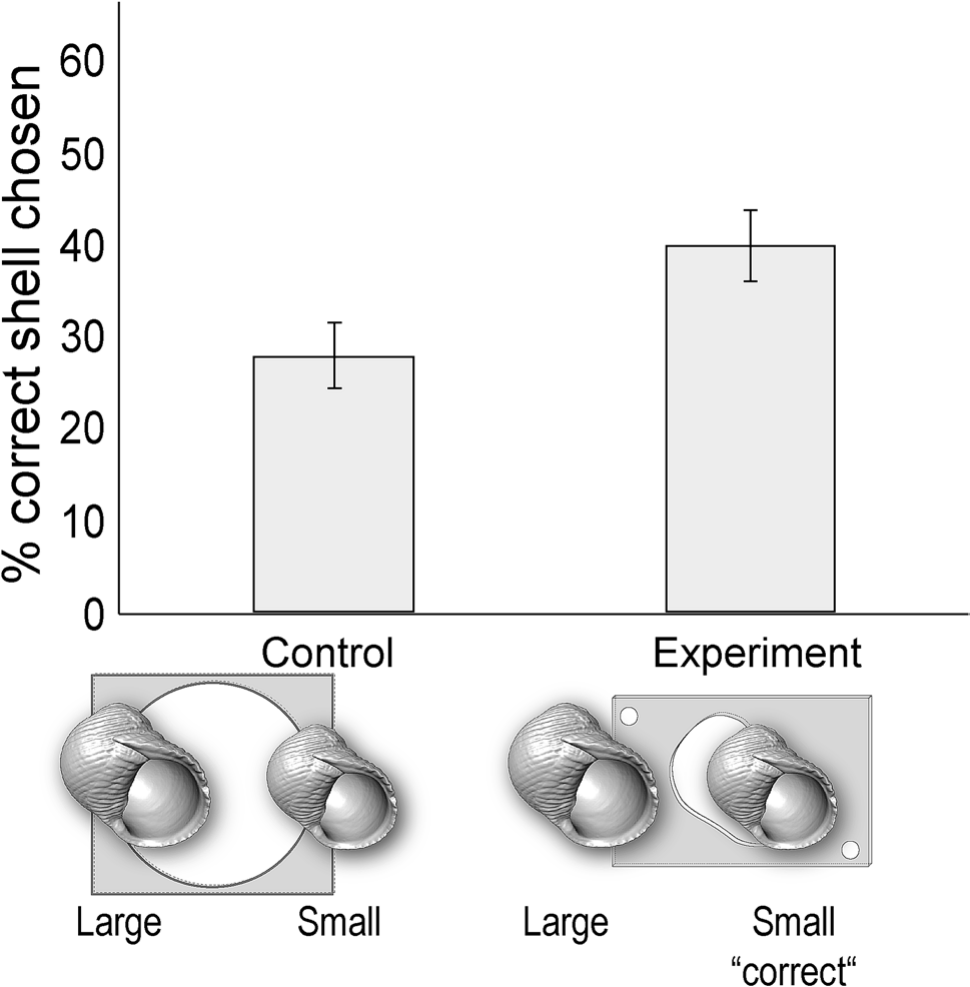
Experiment 1: ‘large’ vs. ‘small’. Percentage of individuals that chose the correct shell (the ‘small’ type), which was necessary to escape in the experimental conditions. *N* = 40 for control and *N* = 40 for experiment. Error bars show standard deviation

**Fig. 4:**
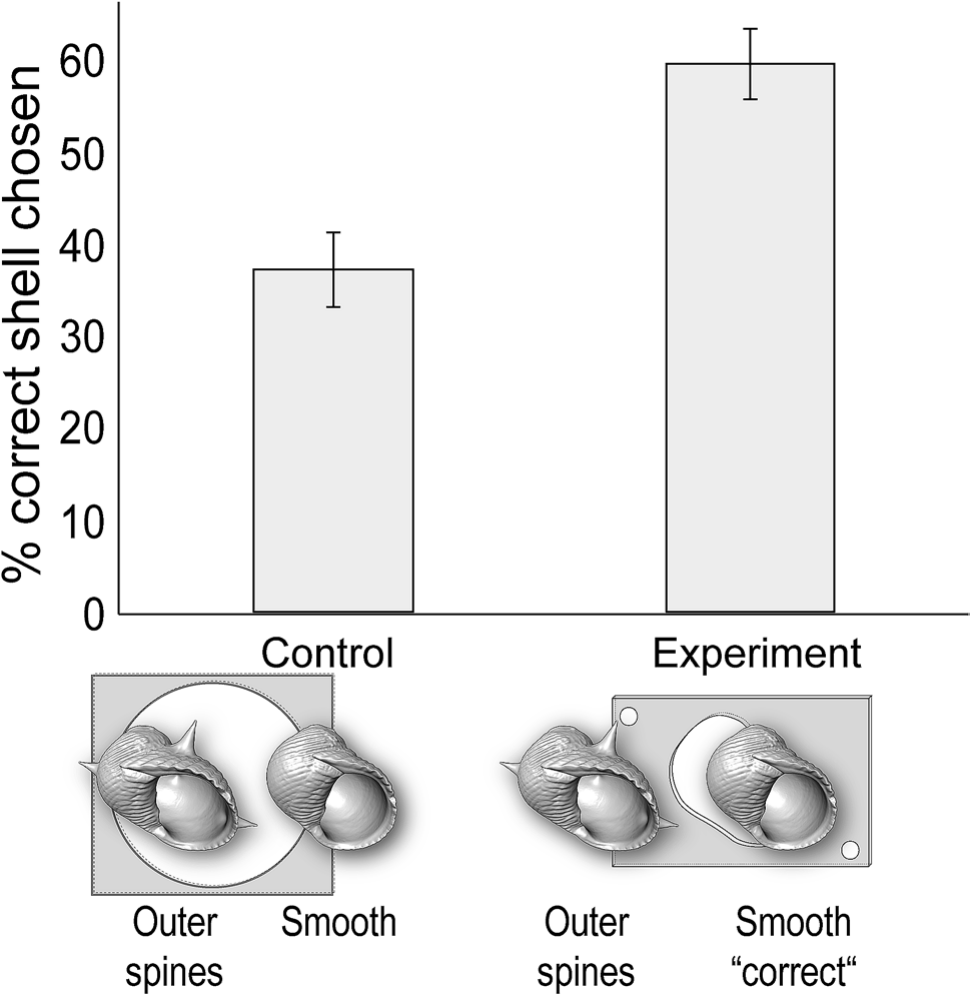
Experiment 2: ‘outer spines’ vs. ‘smooth’. Percentage of individuals that chose the correct shell (the ‘small’ aka ‘smooth’ type), which was necessary to escape in the experimental conditions. *N* = 40 for control and *N* = 40 for experiment. Error bars show standard deviation

**Fig. 5:**
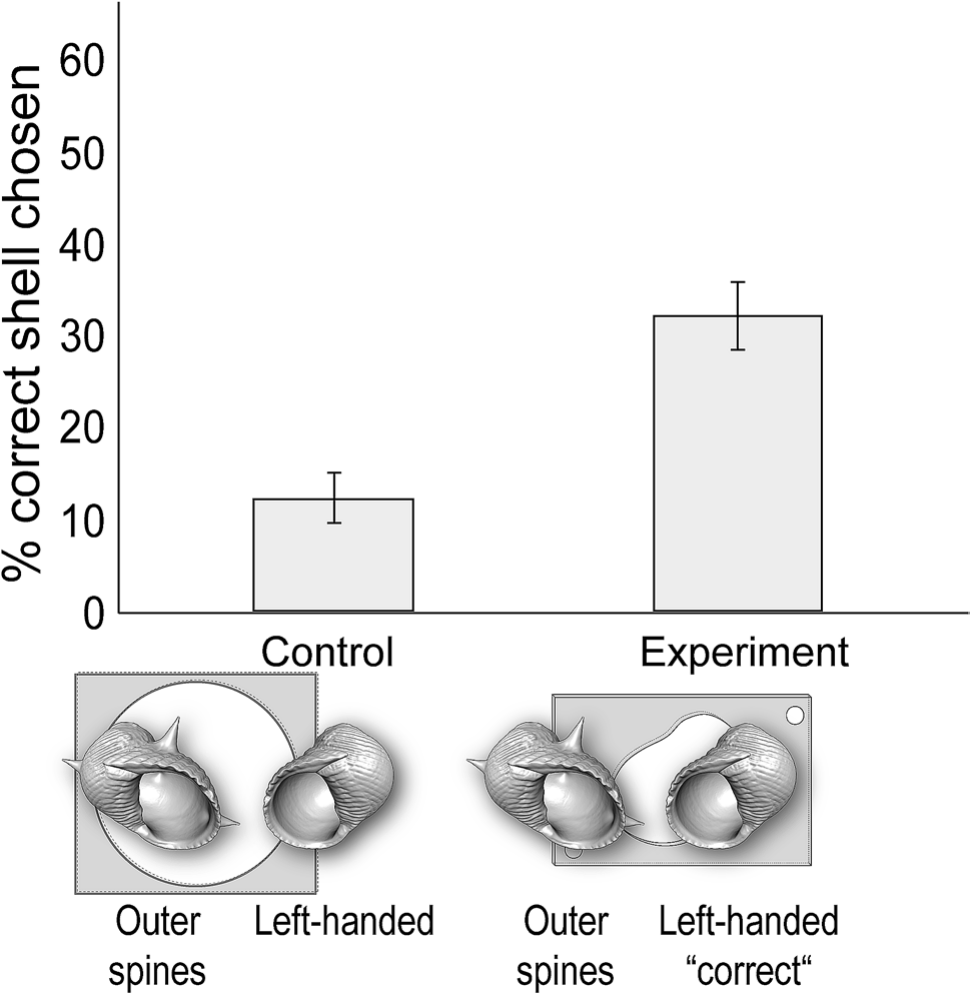
Experiment 3: ‘outer spines’ vs. ‘left-handed’. Percentage of individuals that chose the correct shell (the ‘left-handed’ type), which was necessary to escape in the experimental conditions. *N* = 40 for control and *N* = 40 for experiment. Error bars show standard deviation

**Fig. 6:**
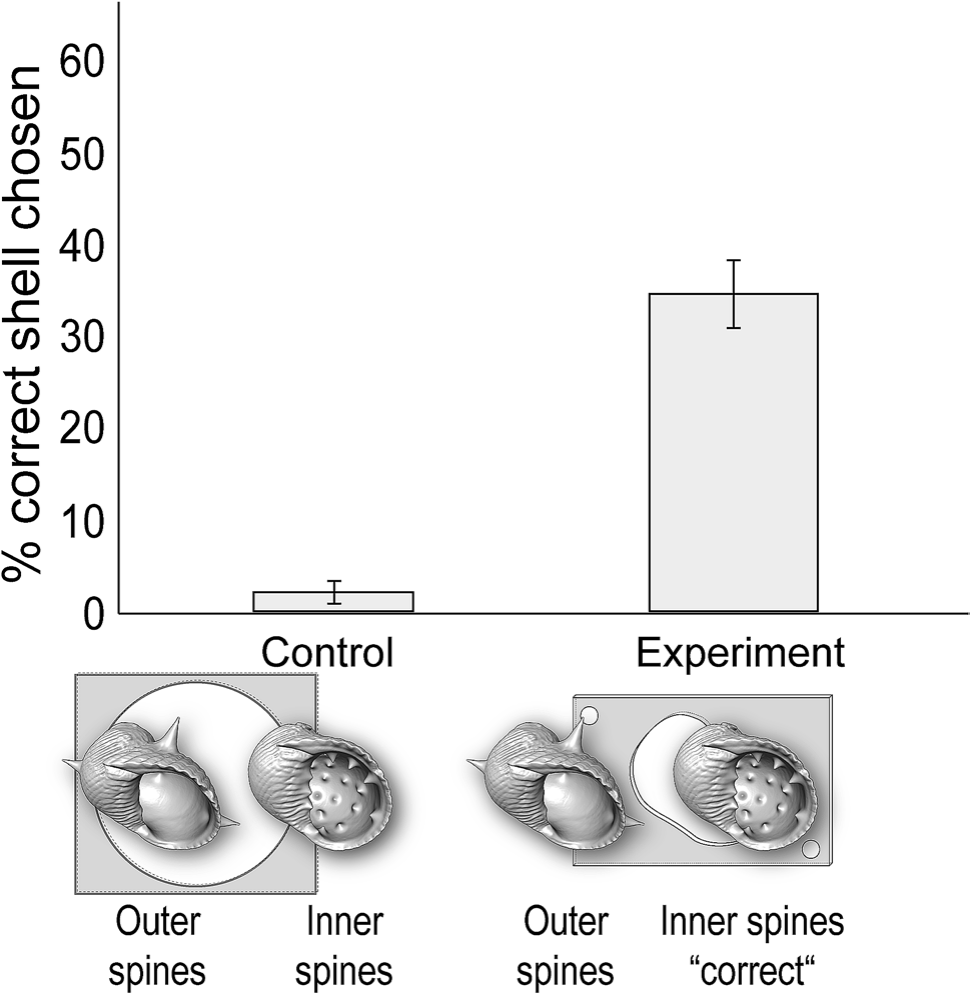
Experiment 4: ‘outer spines’ vs. ‘inner spines’. Percentage of individuals that chose the correct shell (the ‘inner spines’ type), which was necessary to escape in the experimental conditions. *N* = 40 for control and *N* = 40 for experiment. Error bars show standard deviation

Across all four experiments, a substantial proportion of individuals displayed an ability to solve this novel problem of escaping solitary confinement by choosing the shell with the right external architecture (Figs. 3, 4, 5 and 6). For the control condition of each experiment, the choice of shell did not matter, and all crabs in the control conditions immediately left their enclosure in less than 5 min. Interestingly, relative to the control conditions, during the experimental conditions individuals were significantly more likely to choose the correct shells needed to escape in three out of the four experiments (Table S1). In particular, in Experiment 2 (‘outer spines’ vs. ‘small’) individuals chose the correct shell (the ‘small’ shell) significantly more during the experimental compared to the control condition (Chi-square test: *X*^2^ = 4.05, *df* = 1, *p* = 0.0441; Fig. 4). Similarly, in Experiment 3 (‘outer spines’ vs. ‘left-handed’) individuals chose the correct shell (the ‘left-handed’ shell) significantly more during the experimental compared to the control condition (Chi-square test: *X*^2^ = 4.59, *df* = 1, *p* = 0.0322; Fig. 5). This same effect was strongest in Experiment 4 (‘outer spines’ vs. ‘inner spines’), where individuals chose the correct shell (the ‘inner spines’ shell) significantly more during the experimental compared to the control condition (Chi-square test: *X*^2^ = 13.87, *df* = 1, *p* = 0.0002; Fig. 6). Only in one of the experiments (Experiment 1 ‘large’ vs. ‘small’, where the ‘small’ shell was the correct shell) was there not a significant difference between the experimental and the control condition in how often individuals chose the correct shell (Chi-square test: *X*^2^ = 1.40, *df* = 1, *p* = 0.24). Notably, across all four experiments, every individual that did choose the correct shell also ultimately used this shell to successfully escape from its enclosure during the experimental condition.

## Discussion

Novel problems (Gould and Gould 1994; Heinrich 1995; Griffin 2001), which extend beyond what animals have encountered over their own lifetimes or their species’ evolutionary history (Shettleworth 2010; Bueno-Guerra and Amici 2018), can test the limits of cognitive ability (de Waal 2017; Bayne et al. 2019). We found that social hermit crabs were able to solve a challenging novel problem, escaping solitary confinement by choosing the correct shell. Solving this problem was non-trivial, for it required individuals to not only abandon the ineffective shell they started out with, but to switch to the correct shell, which was less preferred and which was also positioned at the far end of their enclosure, away from the escape opening. Furthermore, in the last of our four experiments, the correct shell even contained spines on its inner surface, which would have pushed against crabs’ extremely delicate (Laidre 2007) hind body (pleon), likely making the sheer act of gripping these shells ‘painful’ (Elwood and Appel 2009). Indeed, only a single individual voluntarily utilized the ‘inner spines’ shell in the control condition. Yet, once this otherwise undesirable shell (see Table S1) became necessary to successfully escape, in the experimental condition, then over a third of the animals were willing to choose it. Altogether, the results of our experiments highlight cognitive abilities among invertebrates for solving novel escape problems via extended architecture.

Although many individuals solved the novel problem we posed, many other individuals did not. What accounts for this individual variation (Boogert et al. 2018) in problem-solving success? Differences in motivation between individuals seem unlikely: social hermit crabs are highly motivated to be around conspecifics (Laidre 2010, 2012b, 2014, 2019c), and in their enclosures they were not only isolated from conspecifics but also did not have food or water, making escape extremely motivating. Time constraints also seem unlikely, since after nearly an entire day non-solvers had still not converged on the solution. A more likely explanation for the variation between individuals in their success is differences in the underlying cognitive abilities (Boogert et al. 2018): the problem we posed was novel, and, without past natural selection for solving this problem, individuals could be expected to vary in their skill at solving this problem (Biro et al. 2013; Mann and Patterson 2013). Individual variation in cognition and intelligence is a major current research focus (Boogert et al. 2018), with several key factors, including individuals’ tendency for exploratory behavior and novelty-seeking, helping to explain who is a more successful problem solver. In our study, we did not record the precise time individuals took to solve the problem, though among those that did solve the problem we anecdotally observed substantial variation in how long individuals took, some requiring only minutes and others many hours. Future studies could shed light on this individual variation, exploring what factors (e.g., age, sex or personality) predict whether and how fast individuals reach a solution. The advantage of 3D printed shells in our experiments was in allowing us to precisely control architecture and eliminate any other cues, e.g., chemical cues and color. But in principle, similar follow-up experiments can be done with natural shells.

For individuals that did successfully escape, what underlying mechanisms enabled them to solve this novel navigational problem? Some insight into this question may be gained by comparison of extended architecture with animal tool use (Beck 1980; St Amant and Horton 2008; Shumaker et al. 2011). Paralleling extended architecture, for a behavior to qualify as tool use, an external physical object (not part of the animal’s own body) must be held or manipulated, such that it solves a key problem or serves some adaptive function (Biro et al. 2013). In theory, animals could solve novel tool use problems using a means–ends understanding of the functional properties of tools (Tomasello and Call 1997; St Clair and Rutz 2013), thereby determining in advance which tool is optimal based on the tool’s physical properties in relation to the problem at hand. Alternatively, animals could solve the problem through trial-and-error learning (Hennefield et al. 2018), simply trying different tools until they converge on the correct one, without an appreciation of the relevant properties that make a specific tool right for the problem. Capuchin monkeys, for example, when tested on a novel trap-tube test frequently picked incorrect tools (e.g., tools that did not fit inside the tube or were too short to reach the food) and they also performed actions indicative of limited comprehension (e.g., pushing food into the trap), which overall suggested that they solved this novel tool use problem through trial-and-error learning (Visalberghi and Limongelli 1994; see also Laidre 2008 in baboons).

For our novel escape problem involving extended architecture, crabs could have solved the problem using trial and error, with individuals only trying the alternative shell after repeatedly and ineffectively attempting to exit with the incorrect shell. Alternatively, crabs could have solved the problem by making a deeper, more insightful connection between shell properties (namely, the exterior shell architecture) and the problem (namely, fitting through the escape opening), potentially even anticipating which shell had the properties needed to get out. Individuals of this species can assess shell architecture through multiple modalities, particularly tactile and visual senses (e.g., Laidre 2010, 2013b; Bates and Laidre 2018; Steele and Laidre 2019); and given that terrestrial hermit crabs (*Coenobita* spp.) are able to perceive differences in the outer morphology of the shell they occupy (Sonoda et al. 2012), such perceptual abilities could plausibly have contributed to at least some individuals’ solution. Yet further experiments, with more nuanced tests and detailed video recordings of the entire sequence of behavior individuals display, will be critical to disentangling which specific mechanisms were involved.

Independent of the mechanisms at play, is there any way individuals’ performance and time to solution might be improved on this problem? Both individual learning and social learning (Hoppitt and Laland 2013) could potentially increase how many crabs solve this problem as well as how fast they do so. Indeed, recent studies of brachyuran crabs have provided neural and behavioral evidence of higher-order memory centers, which enable individuals to improve over time on new tasks (Maza et al. 2016). In our experiment, we purposely tested individuals only once, ensuring the problem was entirely novel and hence that their one-time performance indicated an ability, or lack thereof, to solve this problem. Future experiments, however, could test individuals repeatedly on the same problem, determining if individuals learn to choose the right shell more often, getting better over time as the problem becomes more familiar. In addition to individual learning, social learning might also improve performance (Hoppitt and Laland 2013). In our experiment, we explicitly excluded social learning, eliminating possible social cues and forcing individuals to solve the task entirely by themselves. But the addition of social cues in future experiments could have profound impacts, particularly because social hermit crabs are highly sensitive to live conspecific cues (e.g., Laidre 2010) as well as to previously occupied shells (Laidre and Trinh 2014) and leftover by-products like the scent of conspecific death (Valdes and Laidre 2019). Thus, depending on which shell these social cues are paired with (e.g., perfuming the correct versus incorrect shell with conspecific scent), radically different consequences could emerge, either speeding up or slowing down individuals’ problem-solving and solution time. Investigating how social information might influence decisions on this novel escape problem could lead to important evolutionary insights, testing parallels and differences in social learning between invertebrates and vertebrates (Laland and Galef 2009; Seeley 2010; Alem et al. 2016; Loukola et al. 2017; Chittka and Wilson 2019).

In addition to incorporating individual and social learning, our experimental paradigm on extended architecture could profitably be extended further, with a few relatively simple modifications enabling follow-up tests of problem solving at ever finer-grained and more challenging levels (e.g., Alem et al. 2016). For example, simply by widening the variety of shells individuals would need to choose among (from two to many), the choice of the single correct shell could be made even more essential, thereby testing what, if any, foresight individuals display (Bayne et al. 2019). Also, instead of needing to escape just a single enclosure, individuals could be challenged to escape from an inter-connected series of multiple ‘escape rooms’, potentially even requiring that individuals reverse shell choices they made in prior rooms to navigate all the way out of this sequential, multi-step problem. Finally, since social hermit crabs exhibit sophisticated social cognition—forming coalitions to evict others (Laidre 2018c) and also coordinating socially during vacancy chains (Bates and Laidre 2018; Laidre 2019c)—these social skills could be put to the test in cooperative problems (e.g., Beck 1973), where individuals must work together to jointly escape as a team.

Ultimately, further investigations using this experimental paradigm can more deeply explore the cognitive world of invertebrates in the context of extended architecture. Indeed, with a renaissance in the study of arthropod cognition (Seeley 2010; Loukola et al. 2017; Japyassú and Laland 2017; Chittka and Wilson 2019; this special issue), crustaceans have been relatively neglected, despite offering potential model systems that have many advantages: malacostracan crustaceans are highly amenable to manipulative behavioral experiments, including solving a novel escape problem with extended architecture, as in the current study; they offer rich opportunities for comparative tests of cognition in species spanning a remarkable range of different habitats, from deep sea to intertidal to terrestrial (Bracken-Grissom et al. 2013); and they enable detailed studies of brain anatomy, size, and organization (e.g., Harzsch and Hansson 2008; Krieger et al. 2010, 2012b, 2015; Machon et al. 2019; Ramm and Scholtz 2017; Wolff et al. 2017; reviewed in Harzsch and Krieger 2018; Sandeman et al. 1993, 2014; Schmidt 2016; Strausfeld et al. 2020); as well as future opportunities for measuring inter-individual variation in brains, which could link cognitive performance to its underlying physical and neural basis. Continued ‘crustacean cognition’ studies can thus contribute to the burgeoning study of arthropod cognition, as well as to our knowledge of animal cognition broadly.

## Electronic supplementary material

Below is the link to the electronic supplementary material.

Supplementary file1 (DOCX 13 kb)

## Data Availability

All data from this study are available in the electronic supplementary material.

## Footnotes

1 Individuals of this species can be readily removed from their shells (Laidre 2012a, b), which is easy because these shells are architecturally remodeled and thus have very little the crab can grip upon (Laidre 2019b, c). This technique, which has been used in all prior studies, requires only gently gripping the crab’s anterior body parts and then wiggling it free of its shell. After that, the crab can be placed (i.e., “allocated”) to any other shell.

## References

[CR1] Alem S, Perry CJ, Zhu X, Loukola OJ, Ingraham T, Søvik E, Chittka L (2016). Associative mechanisms allow for social learning and cultural transmission of string pulling in an insect. PLoS Biol.

[CR2] Bates KM, Laidre ME (2018). When to socialize: perception of time-sensitive social structures among social hermit crabs. Anim Behav.

[CR3] Bayne T, Brainard D, Byrne RW, Chittka L, Clayton N, Heyes C, Mather J, Olveczky B, Shadlen M, Suddendorf T, Webb B (2019). What is cognition?. Curr Biol.

[CR4] Beck BB (1973). Cooperative tool use by captive hamadryas baboons. Science.

[CR5] Beck BB (1980). Animal tool behavior: the use and manufacture of tools by animals.

[CR6] Biro D, Haslam M, Rutz C (2013). Tool use as adaptation. Philoso Trans R Soc Lond B.

[CR7] Boogert NJ, Madden JR, Morand-Ferron J, Thornton A (2018). Measuring and understanding individual differences in cognition. Philos Trans R Soc Lond B.

[CR8] Bracken-Grissom HD, Cannon ME, Cabezas P, Feldmann RM, Schweitzer CE, Ahyong ST, Felder DL, Lemaitre R, Crandall KA (2013). A comprehensive and integrative reconstruction of evolutionary history for Anomura (Crustacea: Decapoda). BMC Evol Biol.

[CR9] Bueno-Guerra N, Amici F (2018). Field and laboratory methods in animal cognition: a comparative guide.

[CR10] Chittka L, Wilson C (2019). Expanding consciousness. Am Sci.

[CR11] Dawkins R (1982). The extended phenotype: the long reach of the gene.

[CR12] de Waal F (2017). Are we smart enough to know how smart animals are?.

[CR13] Drew MM, Harzsch S, Erland S, Hansson BS (2010). A review of the biology and ecology of the Robber Crab, *Birgus latro* (Linnaeus, 1767) (Anomura: Coenobitidae). Zool Anz.

[CR14] Elwood RW, Appel M (2009). Pain experience in hermit crabs?. Anim Behav.

[CR15] Elwood RW, Neil SJ (1992). Assessments and decisions: a study of information gathering by hermit crabs.

[CR16] Finn JK, Tregenza T, Norman MD (2009). Defensive tool use in a coconut-carrying octopus. Curr Biol.

[CR17] Gould JL, Gould CG (1994). The animal mind.

[CR18] Gould JL, Gould CG (2012). Nature’s compass: the mystery of animal navigation.

[CR19] Griffin DR (2001). Animal minds: beyond cognition to consciousness.

[CR20] Harzsch S, Hansson BS (2008). Brain architecture in the terrestrial hermit crab *Coenobita clypeatus* (Anomura, Coenobitidae), a crustacean with a good aerial sense of smell. BMC Neurosci.

[CR21] Harzsch S, Krieger J (2018). Crustacean olfactory systems: a comparative review and a crustacean perspective on olfaction in insects. Prog Neurobiol.

[CR22] Hazlett BA (1981). The behavioral ecology of hermit crabs. Annu Rev Ecol Syst.

[CR23] Heinrich B (1995). An experimental investigation of insight in common ravens (*Corvus corax*). Auk.

[CR24] Hennefield L, Hwang HG, Weston SJ, Povinelli DJ (2018). Meta-analytic techniques reveal that corvid causal reasoning in the Aesop’s Fable paradigm is driven by trial-and-error learning. Anim Cogn.

[CR25] Hoppitt W, Laland KN (2013). Social learning: an introduction to mechanisms, methods and models.

[CR26] Japyassú HF, Laland KN (2017). Extended spider cognition. Anim Cogn.

[CR27] Krieger J, Sandeman RE, Sandeman DC, Hansson BS, Harzsch S (2010). Brain architecture of the largest living land arthropod, the giant robber crab *Birgus latro* (Crustacea, Anomura, Coenobitidae): evidence for a prominent central olfactory pathway?. Front Zool.

[CR28] Krieger J, Grandy R, Drew MM, Erland S, Stensmyr MC, Harzsch S, Hansson BS (2012). Giant robber crabs monitored from space: GPS-based telemetric studies on Christmas Island (Indian Ocean). PLoS ONE.

[CR29] Krieger J, Sombke A, Seefluth F, Kenning M, Hansson BS, Harzsch S (2012). Comparative brain architecture of the European shore crab *Carcinus maenas* (Brachyura) and the common hermit crab *Pagurus bernhardus* (Anomura) with notes on other marine hermit crabs. Cell Tissue Res.

[CR30] Krieger J, Braun P, Rivera NT, Schubart CD, Müller CHG, Harzsch S (2015). Comparative analyses of olfactory systems in terrestrial crabs (Brachyura): evidence for aerial olfaction?. PeerJ.

[CR31] Laidre ME (2007). Vulnerability and reliable signaling in conflicts between hermit crabs. Behav Ecol.

[CR32] Laidre ME (2008). Spontaneous performance of wild baboons on three novel food-access puzzles. Anim Cogn.

[CR33] Laidre ME (2010). How rugged individualists enable one another to find food and shelter: field experiments with tropical hermit crabs. Proc R Soc Lond B.

[CR34] Laidre ME (2011). Ecological relations between hermit crabs and their shell-supplying gastropods: constrained consumers. J Exp Mar Biol Ecol.

[CR35] Laidre ME (2012). Homes for hermits: temporal, spatial and structural dynamics as transportable homes are incorporated into a population. J Zool.

[CR36] Laidre ME (2012). Niche construction drives social dependence in hermit crabs. Curr Biol.

[CR37] Laidre ME (2013). Foraging across ecosystems: diet diversity and social foraging spanning aquatic and terrestrial ecosystems by an invertebrate. Mar Ecol.

[CR38] Laidre ME (2013). Eavesdropping foragers use level of collective commotion as public information to target high quality patches. Oikos.

[CR39] Laidre ME (2014). The social lives of hermits. Nat History.

[CR40] Laidre ME (2018). Coconut crabs. Curr Biol.

[CR41] Laidre ME, Wellborn GA, Thiel M (2018). Evolutionary ecology of burrow construction and social life. Chapter 11. Life histories.

[CR42] Laidre ME, Bueno-Guerra N, Amici F (2018). Social cognition in the wild: from lab to field in hermit crabs. Field and laboratory methods in animal cognition: a comparative guide.

[CR43] Laidre ME (2019). Life, in a nutshell. Front Ecol Environ.

[CR44] Laidre ME (2019). Private parts for private property: evolution of penis size with more valuable, easily stolen shells. R Soc Open Sci.

[CR45] Laidre ME (2019). Architectural modification of shells by terrestrial hermit crabs alters social dynamics in later generations. Ecology.

[CR46] Laidre ME, Trinh R (2014). Unlike terrestrial hermit crabs, marine hermit crabs do not prefer shells previously used by conspecifics. Crustaceana.

[CR47] Laidre ME, Vermeij GJ (2012). A biodiverse housing market in hermit crabs: proposal for a new biodiversity index. Cuadernos de Investigación UNED.

[CR48] Laidre ME, Patten E, Pruitt L (2012). Costs of a more spacious home after remodelling by hermit crabs. J R Soc Interface.

[CR49] Laland KN, Galef B (2009). The question of animal culture.

[CR50] Lavers JL, Sharp PB, Stuckenbrock S, Bond AL (2020). Entrapment in plastic debris endangers hermit crabs. J Hazard Mater.

[CR51] Lenkei R, Faragó T, Kovács D, Zsilák B, Pongrácz P (2020). That dog won’t fit: body size awareness in dogs. Anim Cogn.

[CR52] Loukola OJ, Perry CJ, Coscos L, Chittka L (2017). Bumblebees show cognitive flexibility by improving on an observed complex behavior. Science.

[CR53] Machon J, Krieger J, Meth R, Zbinden M, Ravaux J, Montagné N, Chertemps T, Harzsch S (2019). Neuroanatomy of a hydrothermal vent shrimp provides insights into the evolution of crustacean integrative brain centers. eLife.

[CR54] Mann J, Patterson EM (2013). Tool use by aquatic animals. Philos Trans R Soc Lond B.

[CR55] Maza FJ, Sztarker J, Shkedy A, Peszano VN, Locatelli FF, Delorenzi A (2016). Context-dependent memory traces in the crab’s mushroom bodies: functional support for a common origin of high-order memory centers. Proc Natl Acad Sci.

[CR56] Ramm T, Scholtz G (2017). No sight, no smell?—brain anatomy of two amphipod crustaceans with different lifestyles. Arthropod Struct Dev.

[CR57] Sandeman DC, Scholtz G, Sandeman RE (1993). Brain evolution in decapod Crustacea. J Exp Zool.

[CR58] Sandeman DC, Kenning M, Harzsch S, Derby CD, Thiel M (2014). Adaptive trends in malacostracan brain form and function related to behavior. Crustacean nervous system and their control of behaviour.

[CR59] Schmidt M, Schmidt-Rhaesa A, Harzsch S, Purschke G (2016). Malacostraca. Structure and evolution of invertebrate nervous systems.

[CR60] Seeley TD (2010). Honeybee democracy.

[CR61] Shettleworth SJ (2010). Cognition, evolution, and behavior.

[CR62] Shumaker RW, Walkup KR, Beck BB (2011). Animal tool behavior: the use and manufacture of tools by animals (revised and updated edition).

[CR63] Sonoda K, Asakura A, Minoura M, Elwood RW, Gunji YP (2012). Hermit crabs perceive the extent of their virtual bodies. Biol Let.

[CR64] St Amant R, Horton TE (2008). Revisiting the definition of animal tool use. Anim Behav.

[CR65] St Clair JJH, Rutz C (2013). New Caledonian crows attend to multiplefunctional properties of complex tools. Philos Trans R Soc Lond B.

[CR66] Steele EP, Laidre ME (2019). Leaf me alone: visual constraints on the ecology of social group formation. Behav Ecol Sociobiol.

[CR67] Strausfeld NJ, Wolff GH, Sayre ME (2020). Mushroom body evolution demonstrates homology and divergence across Pancrustacea. eLife.

[CR68] Tomasello M, Call J (1997). Primate cognition.

[CR69] Valdes L, Laidre ME (2018). Resolving spatio-temporal uncertainty in rare resource acquisition: smell the shell. Evol Ecol.

[CR70] Valdes L, Laidre ME (2019). Scent of death: evolution from sea to land of an extreme collective attraction to conspecific death. Ecol Evol.

[CR71] Vannini M, Cannicci S (1995). Homing behaviour and possible cognitive maps in crustacean decapods. J Exp Mar Biol Ecol.

[CR72] Vermeij GJ (1993). A natural history of shells.

[CR73] Visalberghi E, Limongelli L (1994). Lack of comprehension of cause-effect relations in tool-using capuchin monkeys (*Cebus apella*). J Comp Psychol.

[CR74] Wehner R (2020). Desert navigator: the journey of an ant.

[CR75] Wolff GH, Thoen HH, Marshall J, Sayre ME, Strausfeld NJ (2017). An insect-like mushroom body in a crustacean brain. eLife.

[CR76] Zeil J, Hemmi J (2006). The visual ecology of fiddler crabs. J Comp Physiol A.

