# Supplementary material for: Shells as ‘extended architecture’: to escape isolation, social hermit crabs choose shells with the right external architecture
Source: Anim Cogn. 2020 Aug 8;23(6):1177–87. doi: 10.1007/s10071-020-01419-7 (PMC7700067; doi:10.1007/s10071-020-01419-7)
Supplement: Supplementary file 1 — Supplementary file1 (DOCX 13 kb) [file 10071_2020_1419_MOESM1_ESM.docx]

**Table S1.** Proportions of animals’ shell choices after 20 h in the control and experimental conditions for each experiment; and Chi-square tests for Experiments 1 to 4. In the Results section, as well as here in this table, Chi-squared tests compared the choice of the correct shell between the experimental condition versus the control condition. This table also includes additional Chi-squared tests that compared the choice of the “correct” shell in the experimental condition as well as in the control condition versus an arbitrary threshold of 50% each.

| **Shell type** | **Control 1** | | **Experiment 1** | | |  |  |
| --- | --- | --- | --- | --- | --- | --- | --- |
| Large | 29 | | 24 | | |  |  |
| Small “correct” | 11 | | 16 | | |  |  |
| **Shell type** | **Control 2** | | **Experiment 2** | | |  |  |
| Outer spines | 25 | | 16 | | |  |  |
| Smooth “correct” | 15 | | 24 | | |  |  |
| **Shell type** | **Control 3** | | **Experiment 3** | | |  |  |
| Outer spines | 35 | | 27 | | |  |  |
| Left-handed “correct” | 5 | | 13 | | |  |  |
| **Shell type** | **Control 4** | | **Experiment 4** | | |  |  |
| Outer spines | 39 | | 26 | | |  |  |
| Inner spines “correct” | 1 | | 14 | | |  |  |
|  |  | | | |  |  |  |
| **Experiment** | **Control condition versus arbitrary threshold** | | | | **Experimental condition**  **versus**  **arbitrary threshold** | | **Experimental condition**  **versus**  **control condition** |
| **Experiment 1**  **(‘large’ vs. ‘small’)** | | X^2^ = 4.27, df = 1, p = 0.0389 | | X^2^ = 0.81, df = 1, p = 0.37 | | | X^2^ = 1.40, df = 1, p = 0.24 |
| **Experiment 2**  **(‘outer spines’ vs. ‘small’)** | | X^2^ = 1.27, df = 1, p = 0.2598 | | X^2^ = 0.81, df = 1, p = 0.37 | | | X^2^ = 4.05, df = 1, p = 0.0441 |
| **Experiment 3**  **(‘outer spines’ vs. ‘left-handed’)** | X^2^ = 13.09, df = 1, p = 0.0003 | | | | X^2^ = 2.53, df = 1, p = 0.11 | | X^2^ = 4.59, df = 1, p = 0.0322 |
| **Experiment 4**  **(‘outer spines’ vs. ‘inner spines’)** | X^2^ = 23.31, df = 1, p << 0.0001 | | | | X^2^ = 1.84, df = 1, p = 0.17 | | X^2^ = 13.87, df = 1, p = 0.0002 |
